# Reproduction of Bones and Joints, after Their Removal in Cases of Whitlow

**Published:** 1857-12

**Authors:** W. H. Toland

**Affiliations:** San Francisco, California


*Reproduction of Bones and Joints, after their Removal in cases of Whitlow.* By W. H. TOLAND, M. D., of San Francisco, California.

*Paronychia or Whitlow: its Consequences and Treatment.*—Although this is an exceedingly painful disease, and frequently destroys the parts affected, it has received but little attention from the profession, because it rarely endangers life. There are four varieties of Whitlow:

1st. That seated between the epidermis and cutis vera.

2d. That situated in the sub-cutaneous cellular tissue.

3d. That occupying the sheaths of the tendons.

4th. That seated between the periosteum and bone.

Every phlegmonous inflammation of the fingers and toes belongs to one of the forms of paronychia above specified. Every variety of this disease is excessively painful. The first three are speedily removed by a free incision; but the fourth is a much more serious affection. Even when treated early and properly, it frequently causes a destruction of the bone, in consequence of a separation of its periosteum; pus forms between that membrane and the bone; the former is detached, and the latter loses its vitality, and if not removed will be destroyed.

As the bone is formed by the periosteum, if the latter be not removed, and the soft parts are kept extended, the bones as well as the joints are re-produced, and the part restored to its former usefulness. It has long been known that the bone will be re-produced, provided the periosteum remains, and we only claim to have made the discovery that the joints are also, by proper management, restored, even under the most unfavorable circumstances. Whether they are as perfect as the original, possessing cartilages, ligaments, and synovial fluid, we are unable to say; but we know that they possess motion, and sufficient strength to perform the function of the most perfect joints.

James Clarke, a porter, now of Columbia, Tuolumne county, consulted us respecting the propriety of having the middle finger of the right hand amputated. He had been treated by a good surgeon, who thought its removal necessary.

As only the third, and half of the second phalanx were diseased, and believing that they would be re-produced, we advised their removal, so that if even there was no motion in the first joint, the finger would still be useful. An incision was made from the first joint, extending laterally, under the nail, to the same point on the opposite side. The soft parts were detached from the denuded bone, and the second phalanx divided with bone forceps about a quarter of an inch from the second joint. The wound was closed by the interrupted suture, and the soft parts retained in a proper position, by the application of splits and a bandage.

In four weeks, to my astonishment, *the bones were not only restored*, but also *a perfect joint was formed.* The finger is now as strong, and the motion of the newly formed joint as perfect as the original. Being a porter, he injudiciously used the finger before the bones were perfectly solidified, and a slight lateral curvature was produced; but in every other respect the finger is as perfect and useful as before the occurrence of the accident.

McGowen, now employed at Newland's stables, on Kearney-street, was advised, in January, 1857, by his physician, to have the forefinger of the right hand amputated at the second joint, and he came to our office for the purpose of complying with his instructions. Instead of amputating the finger, the phalanges were removed, and the wound dressed as in the former case In a few days, although requested to visit the office until cured, he renewed his occupation, and we lost sight of him entirely. On the 10th of June his finger was examined, and we found the second joint perfect, and the entire phalanx restored. The last phalanx, in consequence of the soft parts being allowed to contract, is short, although a joint exists, and the finger is as strong and useful as before the operation.

Shannon, a cooper, living at No. 6 Jackson-street, had a whitlow of the first forefinger, involving only the last phalanx, which was removed at the joint by a lateral incision. He resumed his business in a few days after the operation, and the finger was not examined for several months. The newly formed bone is shorter than the original one, although the motion of the joint is perfect, and the finger in every respect as serviceable as before.

Other cases might be given, if these were not considered sufficient, to establish the point in question.

Cullens, who lives on Stevenson-street, between First and Second-streets, had suffered for three months before he became my patient, from a whitlow, involving the whole of the right thumb. Free incisions had been made, without affording relief. The thumb was enormously enlarged, the first and second phalanges were denuded, and the flexor tendon near the extremity destroyed. Notwithstanding its excessively diseased condition, we determined to remove the bones, believing they would be re-produced, although we were confident that the thumb would not be as perfect as if he had received proper attention at an earlier period.

An incision was made upon the upper side of the thumb near the location of the extensor tendon, and carried upwards under the nail, and both phalanges removed. This operation was performed about the first of

April, and in six weeks the soft parts were healthy, and the bones and joints re-produced. Although he has but little control over the last phalanx, in consequence of the destruction of the tendon, the thumb is nearly as long as its fellow of the opposite hand. It presents a natural appearance, and is improving daily, both in strength and motion.

A gentleman, living near Redwood city, had suffered for several weeks from a similar disease, and was operated upon on the 15th of April, 1857. In his case a lateral incision was made, and both phalanges of the right thumb were excised. This case was progressing much more favorably than that of Cullen's, when he left the city, two weeks after the operation. He has not been heard from since, although no doubt is entertained of the entire restoration of both the bones and articulations.

In 1853, a carpenter was admitted into the State Marine Hopital with a compound comminuted fracture of the great toe. After remaining two months, and finding that union had not taken place, and that the bones were diseased, instead of amputating the toe, we removed the bones, and in a short time they were restored, although we were not aware of the re-production of the articulations.

Blaisdell, who then lived near Oakland, had the great toe of the left foot injured by a large piece of timber. When examined, we found the second phalanx diseased, although the foot was healthy. In December, 1856, an incision was made upon the internal side, and extended from the articulation with the metatarsal bone to the extremity under the nail. Both phalanges were removed, and the wound closed by the interrupted suture. In four weeks from the time the operation was performed, the bones were re-produced, and the patient could wear a boot without inconvenience. The articulations appear to be perfect, although the last phalanx was not diseased and was removed with the periosteum. This can only be accounted for by supposing that the periosteum detached from the second phalanx produced a sufficient quantity of osseous matte for the restoration of both. Mr. Blaidsell is now employed on Bird Island, and suffers no inconvenience from the injury.

In removing the diseased bones from either the fingers or toes, lateral incisions should be made to avoid the tendons; and the soft parts, until bony matter is deposited, should be supported by pasteboard splits, applied so loosely as not to produce pain. If they be found inconvenient, the other fingers or toes may serve as splints. Before the bones are fully developed, the member should be flexed occasionally, although the precaution was not taken in some of the most satisfactory cases given above. In Cullen's case, in consequence of the destruction of the tendon, an effort was made to produce anchylosis of the first joint, by the application of splints, but without success.

Many operations of a similar character have been performed in the United States Marine Hospital, but not being enabled to furnish the results, they have been omitted. Most of the cases cited can be examined by the incredulous, and the above statements verified. The result of these experiments is not only interesting but extremely useful. Useful because by the loss of a thumb a laboring man may be deprived of the means of pursuing the only occupation with which he is acquainted, and by which alone he can support himself and family. By the loss of the great toe, the strength of the foot is impaired, the gait is rendered unsteady, and locomotion fatiguing. Interesting, because it establishes the efficiency of the restorative powers of the human system more fully and unquestionably than anything that has heretofore been observed; and when assisted and directed by those who, from greater skill and experience, are more competent than ourselves, to act efficiently, results more brilliant than we can now imagine may be realized.—*Charleston Medical Journal.*

